# Cathodoluminescence and tip-plasmon resonance of Bi_2_Te_3_ triangular nanostructures

**DOI:** 10.1371/journal.pone.0291251

**Published:** 2024-01-19

**Authors:** Qigeng Yan, Siyuan Wang, Kuiwen Guan, Xiaojin Guan, Lei He

**Affiliations:** 1 Department of Physics, Baoding University, Baoding, Hebei, China; 2 Department of Physics, University of Arkansas, Fayetteville, Arkansas, United States of America; 3 Department of Science and Research, Baoding University, Baoding, Hebei, China; Universiti Brunei Darussalam, BRUNEI DARUSSALAM

## Abstract

Bi_2_Te_3_, as a topological insulator, is able to support plasmonic emission in the visible spectral range. Thin Bi_2_Te_3_ flakes can be exfoliated directly from a Bi_2_Te_3_ crystal, and the shape of Bi_2_Te_3_ flakes can be further modified by focused ion beam milling. Therefore, we have designed a Bi_2_Te_3_ triangular antenna with distinct tip angles for the application of plasmonic resonance. The plasmonic emission of the Bi_2_Te_3_ triangular antenna is excited and investigated by cathodoluminescence in the scanning electron microscope. Enhanced tip plasmons have been observed from distinct tips with angles of 20º, 36º, 54º, 70º, and 90º, respectively. Due to the confinement of geometric boundaries for oscillating charges, the resonant peak position of tip plasmon with a smaller angle has a blue shift. Moreover, the dependence of plasmonic behavior on the excitation position has been discovered as well. This research provides a unique approach to fabricate Bi_2_Te_3_ nanostructures and manipulate the corresponding plasmonic properties.

## Introduction

Three-dimensional topological insulators (3D TIs) have been an attracting research topic due to their unique physical properties in recent years [1–4]. As one of commonly investigated 3D TIs, though Bi_2_Te_3_ is well known as a thermoelectric material [5, 6], it is also reported that Bi_2_Te_3_ nanostructures are capable of supporting surface plasmons (SPs) [7–11]. Due to the existence of metallic surface states, collective oscillations of surface charges and propagating electromagnetic waves can be generated across the interface between the Bi_2_Te_3_ and a dielectric medium [7]. Nowadays, most correlated SP applications are limited to noble metals [7, 8], such as Au and Ag, so the development of Bi_2_Te_3_ nanostructures is meaningful to enrich the option of plasmonic materials. The dispersive property of SPs relates to the complex dielectric function of the plasmonic material [12]. In other words, the real part of the permittivity (ε_r_) of Bi_2_Te_3_ has to be negative to generate plasmonic resonances, while the imaginary part of the permittivity (ε_i_) relates to the absorption of electromagnetic radiation. From both experimental and theoretical studies of Bi_2_Te_3_, it is indicated that ε_r_ and ε_i_ agree with the Kramers-Kronig relation [8, 13, 14]. Consequently, based on the strong oscillating strength of the inter-band transition for Bi_2_Te_3_, the plasmonic resonance is expected to be excited in the visible spectral range, since ε_r_ is negative from 240 nm to 798 nm [8]. Meanwhile, ε_i_ increases gradually from the visible range to the infrared range, and reaches a peak around 1120 nm, indicating a higher damping effect of the plasmonic resonance in the relative area. Concluded by Toudert et al. and Yin et al. [14], results of the plasmonic quality factor and the plasmonic figure of merit (FOM) indicate that Bi_2_Te_3_ is possible to have a better plasmonic stability across the whole visible range than Au and Ag [13, 14].

The enhanced edge and bulk plasmon modes caused by the localized charge oscillation at edges or between the center and edges in Bi_2_Te_3_ nanoflakes are observed and reported [7–11]. Especially, due to the strong confinement on the incident beam spot size, electron-beam related spectroscopies, such as electron energy loss spectroscopy (EELS) and cathodoluminescence (CL), can be employed to excite Bi_2_Te_3_ nanostructures [7, 10]. Therefore, due to the gradually shrinking size in geometry [15], sharp tips generated by the intersection of edges are possible to be confirmed to support enhanced plasmonic resonance as well. Because of the outstanding focusing and propagating abilities for SP resonances, plasmonic nanostructures with metallic tips, wedges, or strips have been proposed previously [16–24]. Compared to common flat or curved geometries, structures corners or tips have more potential to be included into plasmonic devices [25–27]. Possible applications for the tip-like structure with a strong confined electromagnetic field include optical waveguide [16–19], SP biosensors or detection [20, 21], and nanoemittors [22]. Hence, it is worthy to investigate the plasmonic emission in Bi_2_Te_3_ tips.

In this work, we propose a Bi_2_Te_3_ triangular antenna with three different angles for the investigation of SP resonance at the tip area. The traditional solvothermal method is widely used to synthesize Bi_2_Te_3_ nanostructures [7]. However, in this research, Bi_2_Te_3_ flakes are mechanically exfoliated out of a bulk crystal, Therefore, the structure and size can be further designed and refined by focused ion beam (FIB) milling [28]. Artificial plasmonic nanostructures composed of closing boundaries are expected to exhibit unique optical enhancement in the visible range [29–31]. Then, Bi_2_Te_3_ tips are interacted with the focused electron beam to excite the plasmonic resonance. To avoid the deposition of carbon or the oxidation in air, all the milling and characterization processes are conducted inside the same vacuum chamber [32]. Plasmonic properties based on the tip size, excitation position and band-pass wavelength are acquired and investigated by cathodoluminescence.

## Materials and methods

**Fig 1:**
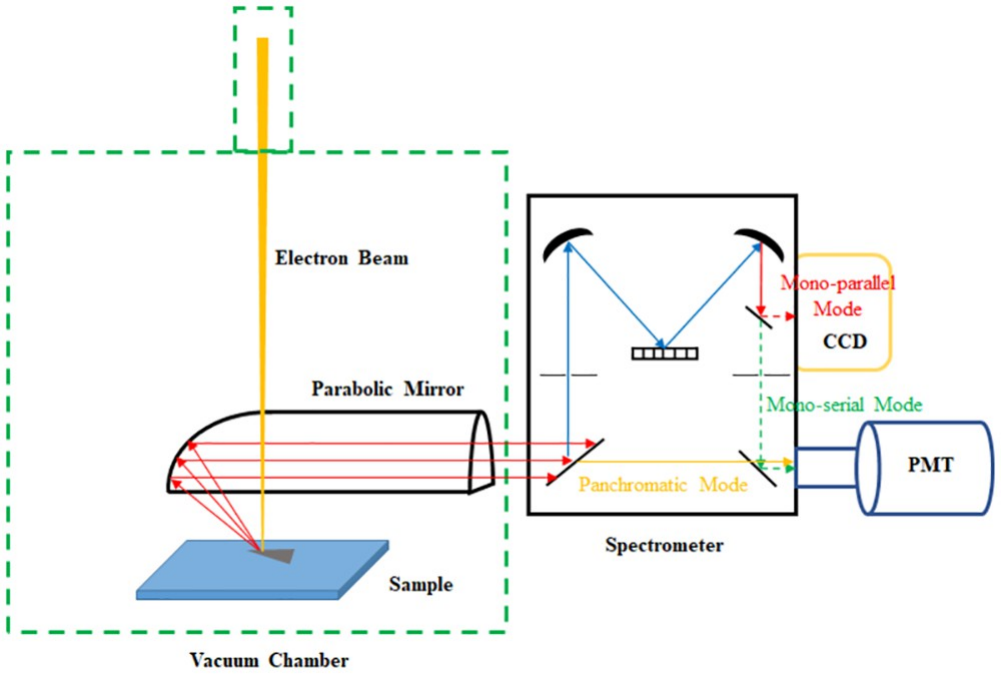
Schematic of the CL characterization process accompanied inside a SEM chamber.

Bi_2_Te_3_ triangular structures were prepared following the process of Ref. [10]. The Si substrate with a 100 nm SiO_2_ top layer was first cleaned by hydrogen fluoride (HF), acetone and isopropyl alcohol (IPA) to reduce surface contamination and oxidation. Then, Bi_2_Te_3_ flakes were mechanically exfoliated from a Bi_2_Te_3_ crystal (2D Semiconductors, 99.9999% purity) onto the Si substrate. Bi_2_Te_3_ flakes with a smooth surface was milled by FIB in a FEI Nova Nano SEM450 system (Hillsboro, OR, USA) with a Ga^+^ ion-beam source. Irregular edges on the flake were removed by FIB to form a right triangular shape with different tips, including 20°, 36°, 54°, 70°, and 90° tips. The average thickness of the antenna is 270 nm directly measured after tilting the stage to 52°. Surface morphology images, energy-dispersive X-ray (EDX) spectra and cathodoluminescence were acquired in a same scanning electron microscope (SEM) system. The SEM image and EDX map were obtained by scanning a focused electron beam with 15 KeV beam energy. On the other hand, the sample was excited by the electron beam energy of 30 KeV for all CL characterizations. Compared to the conventional photoluminescence, the application of CL could provide a better spatial resolution to observe the optical properties of nanostructures, so it is a suitable technique to investigate Bi_2_Te_3_ triangular antennas with a shrinking shape at tips. The CL signal was acquired by inserting an Al parabolic mirror 0.5 mm above the sample and transferred to a Gatan MonoCL4 spectrometer. As presented in Fig 1, the CL panchromatic mode was utilizing a photomultiplier tube (PMT) with a band-pass wavelength from 300 nm to 900 nm as the detector. Although the measurement by PMT could receive a better sensitivity, it also suffers from a slow working speed. As a compensate, the CL monochromatic function was obtained by a CCD camera with a wider band-pass range, from 250 nm to 1100 nm, after the reflection by lens and gratings.

## Results and discussion

**Fig 2:**
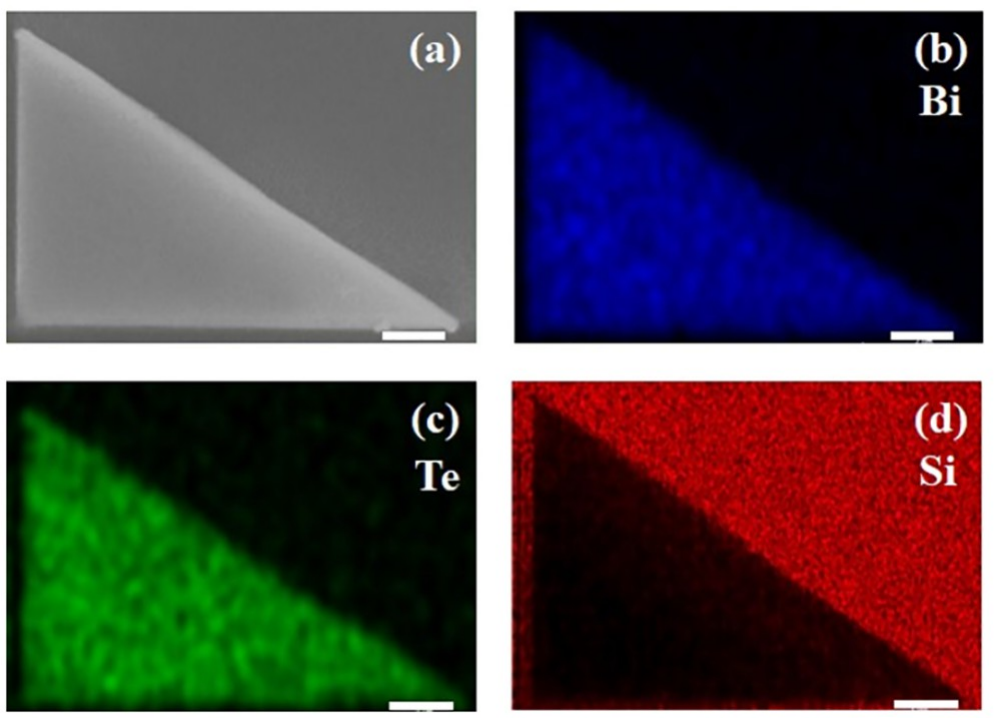
(a) SEM image and (b-d) EDX maps of the Bi_2_Te_3_ triangular nanoflake with three different angles on a Si substrate. All scale bars represent 1 μm.

Size and geometry of a Bi_2_Te_3_ triangular flake is shown by the SEM image in Fig 2(a), the Bi_2_Te_3_ triangle contains three edges with different length, which are 7.60 μm, 6.30 μm, and 4.25 μm, separately. Therefore, the tips enclosed by edges are 90°, 56°, and 34°, respectively. Unlike other plasmonic structures patterned by FIB milling, such as metallic bullseye nanoresonators [32], the Bi_2_Te_3_ triangular antenna exhibits a clean surface and low roughness on edges. Tips are separated far away enough, in the order of micrometer, to minimize the interaction of localized field between adjacent sharp tips [22]. Even though the lateral size of the Bi_2_Te_3_ triangular antenna is large compared to the expected wavelength of surface waves, the width of sharp tips can be lower than 100 nm. The tips with sub-wavelength dimension are possible to generate enhanced plasmonic fields. Fig 2(b)–2(d) presents the EDX elemental maps of three major components in the sample, which are Si, Bi, and Te. Inside the triangular area, Bi (blue) and Te (green) are the dominant elements, however, Si (red) provides less contrast. Whereas, Si is the most obvious element outside the triangular area. The clear division between Bi_2_Te_3_ and Si indicates that the collision of Ga^+^ ions does not affect the distribution of materials. The scattering effect caused by the re-deposited Bi_2_Te_3_ particles after FIB milling can be ignored.

**Fig 3:**
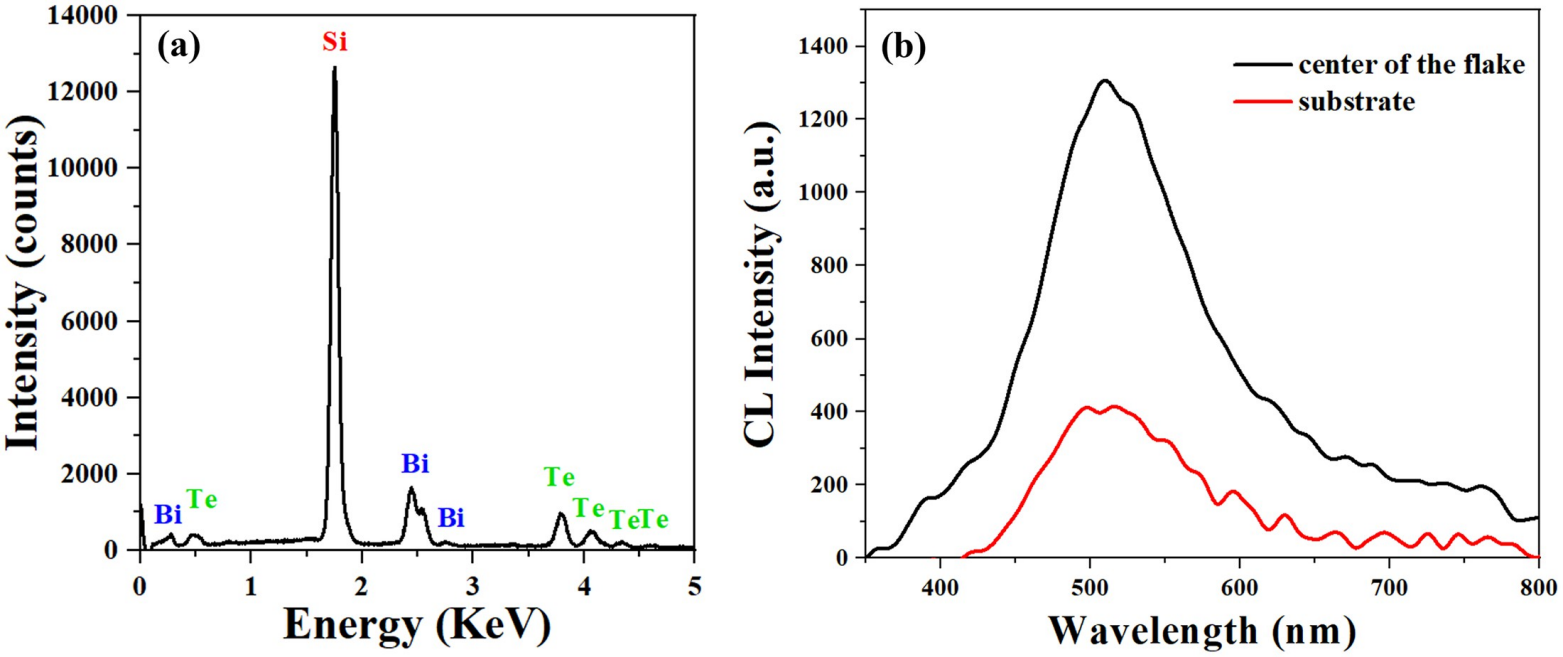
(a) EDX spectrum of the same Bi_2_Te_3_ flake; (b) CL spectra collected from the center of the Bi_2_Te_3_ flake and the substrate.

In Fig 3(a), the EDX spectrum presents distinct peaks of Bi, Te, and Si. The atomic percentage ratio of Bi and Te is 0.679, as suggested by the quantified EDX values. By localizing a focused e-beam at steady spots, we compare the CL emission from the triangular Bi_2_Te_3_ flake and the substrate. As shown in Fig 3(b), the black curve presents a CL spectrum acquired from the center of the Bi_2_Te_3_ flake, and the red line corresponds to a CL spectrum collected from the substrate without Bi_2_Te_3_. The electron beam spot size is focused around 5 to 10 nm. The Monte Carlo simulation (CASINO Monte Carlo simulation ver 2.51, Dr. Drouin, University of Usherbrooke, Canada) of electron trajectories (S1 and S2 Figs) reveals that there is no obvious beam broadening inside the Bi_2_Te_3_. We claim that the CL emission from the substrate mainly originates from the SiO_2_ layer [33]. For both spectra, peak positions with the maximum intensity are located at 510 nm, while the maximum CL intensity from the Bi_2_Te_3_ flake is two times higher than that from the substrate due to the plasmonic enhancement effect [34]. Relative results reveal that Bi_2_Te_3_ flakes provide a broad-range emission peak, mainly bulk and edge plasmons, across the visible spectral range [7, 8]. Moreover, for the electron-beam excitation in this case, both incident electrons and secondary electrons interact with the Bi_2_Te_3_ and substrate, generating localized surface plasmon waves. Therefore, as a plasmonic material, Bi_2_Te_3_ flakes or structures increase the absorption efficiency by the electron-material interaction. The total emission efficiency will be improved by the localized electric field. As a result, the CL intensity collected from a Bi_2_Te_3_ flake is clearly stronger.

**Fig 4:**
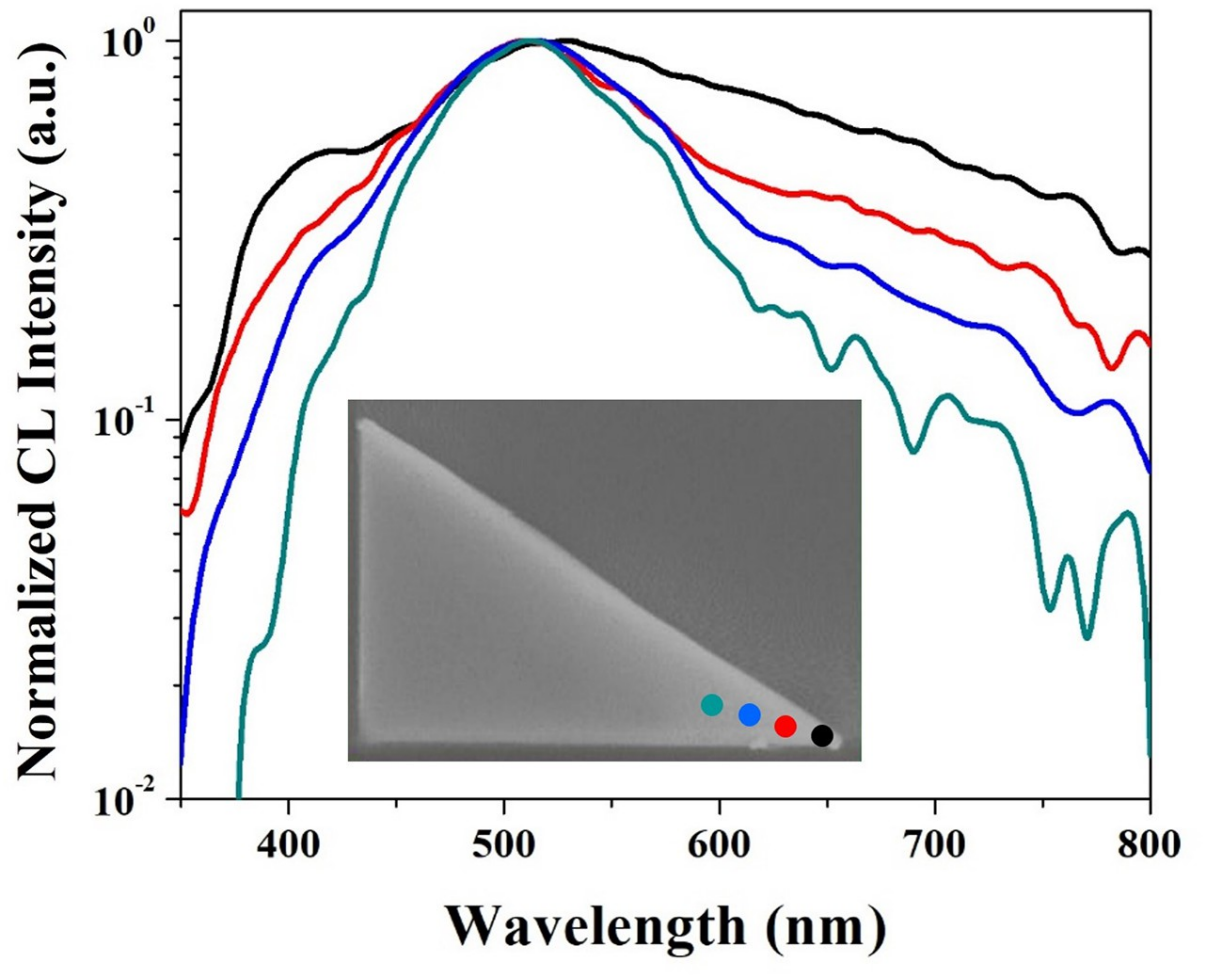
CL spectra excited at four spots along the angle bisector of the 36° tip. Excitation locations are shown in the inserted SEM image. The color of each curve matches with the color of excitation spots.

In order to investigate more details of the plasmonic resonance at the tip, CL spectra have been applied based on its advanced spatial resolution. Fig 4 illustrates the normalized spectra excited along the angle bisector of a 36° tip. Four excitation locations are indicated in the inset SEM image. The color of each excitation spot matches with the color of corresponding the spectrum. The separation between two adjacent spots is about 350 nm. An increasing linewidth has been obtained as the beam location moving towards the tip end, indicating the emission of gradual arising SP modes when the excitation spot is closer to the boundary. A unique bulge is observed on the dark curve around 400 nm. It could be attributed to the tip plasmon, due to the interaction of oscillating charges between two intersecting edges. This plasmonic emission becomes more obvious when the beam excites at the tip end.

**Fig 5:**
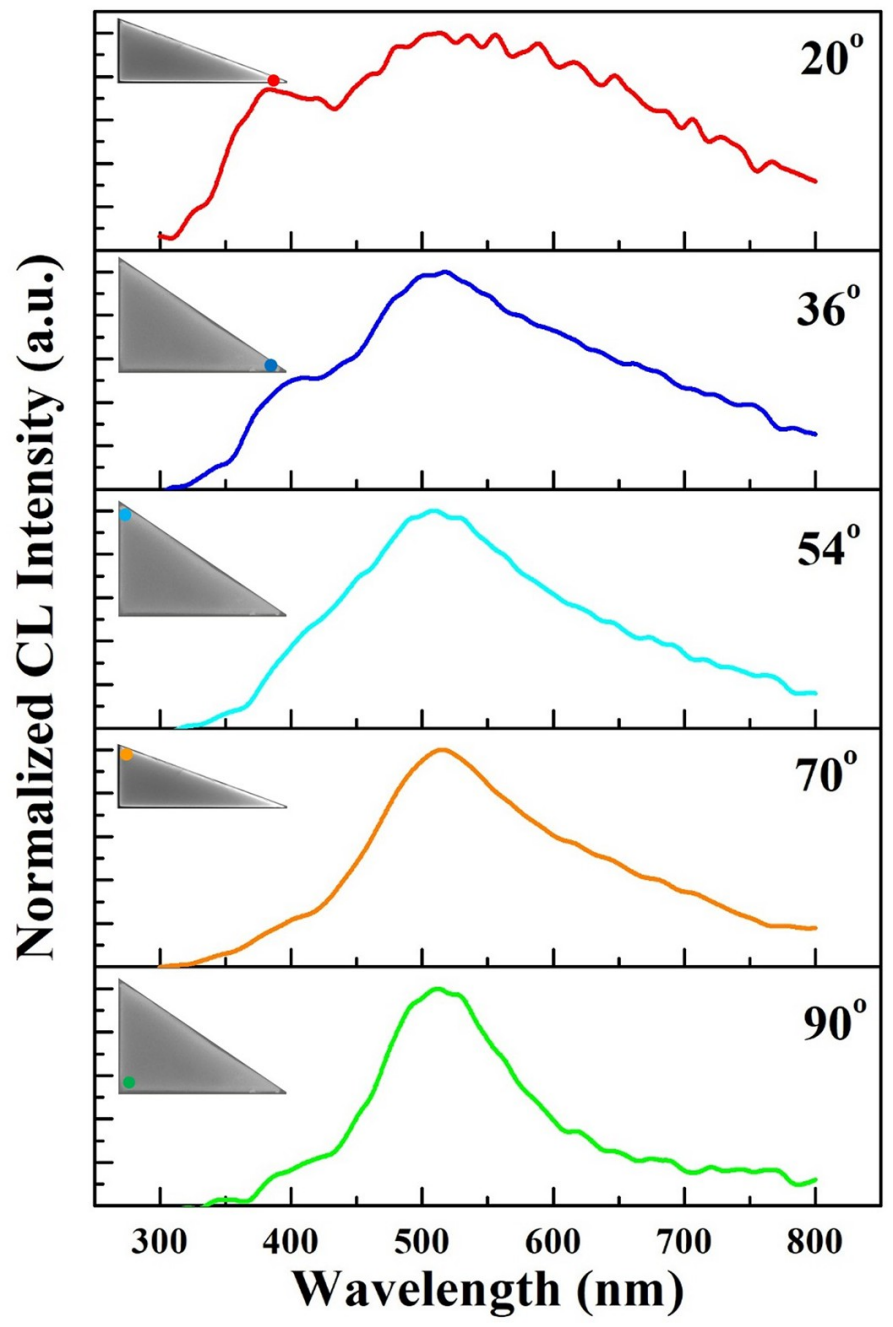
Normalized CL spectra acquired from Bi_2_Te_3_ nanotips with different tip angles. Excitation locations are indicated as inserted SEM images.

To further investigate the correlation between plasmonic resonance and tip size, we locate the focused electron beam exactly at the tips to excite CL emission. Corresponding CL spectra of five distinct tips (90°, 70°, 54°, 36° and 20°) are presented in Fig 5, with excitation spots labeled as insets. A CL emission peak centered at 510 nm is observed when the electron beam is localized at the tip with a right angle (green curve). It possesses a similar line shape with the spectrum from the center of the flake. Due to the relative strong background luminescence, the expected plasmonic resonance of the tip plasmon is not obvious for the right angle. On the other hand, it is showing that the spectra from acute angles with quite different plasmonic behavior. Apparently, the linewidth of these curves is higher than the spectrum from the right angle. For the long-wavelength side, the confinement of edges dominates [7]. Edge plasmons gradually grow up for sharper tips, leading to a higher slope at the long-wavelength side. For structures with confined boundaries, such as tips or edges, the surface charge density is preferred to accumulate at these regions, leading to enhanced plasmonic modes [31, 35, 36]. Meanwhile, it indicates that a strong collective tip plasmon emission has been excited due to the resonance between edges and sharp tips. The width and intensity of such plasmonic resonance increases as the tip-angle decreasing. As a result, the tip plasmon emission from the 20° angle becomes the most explicit.

**Fig 6:**
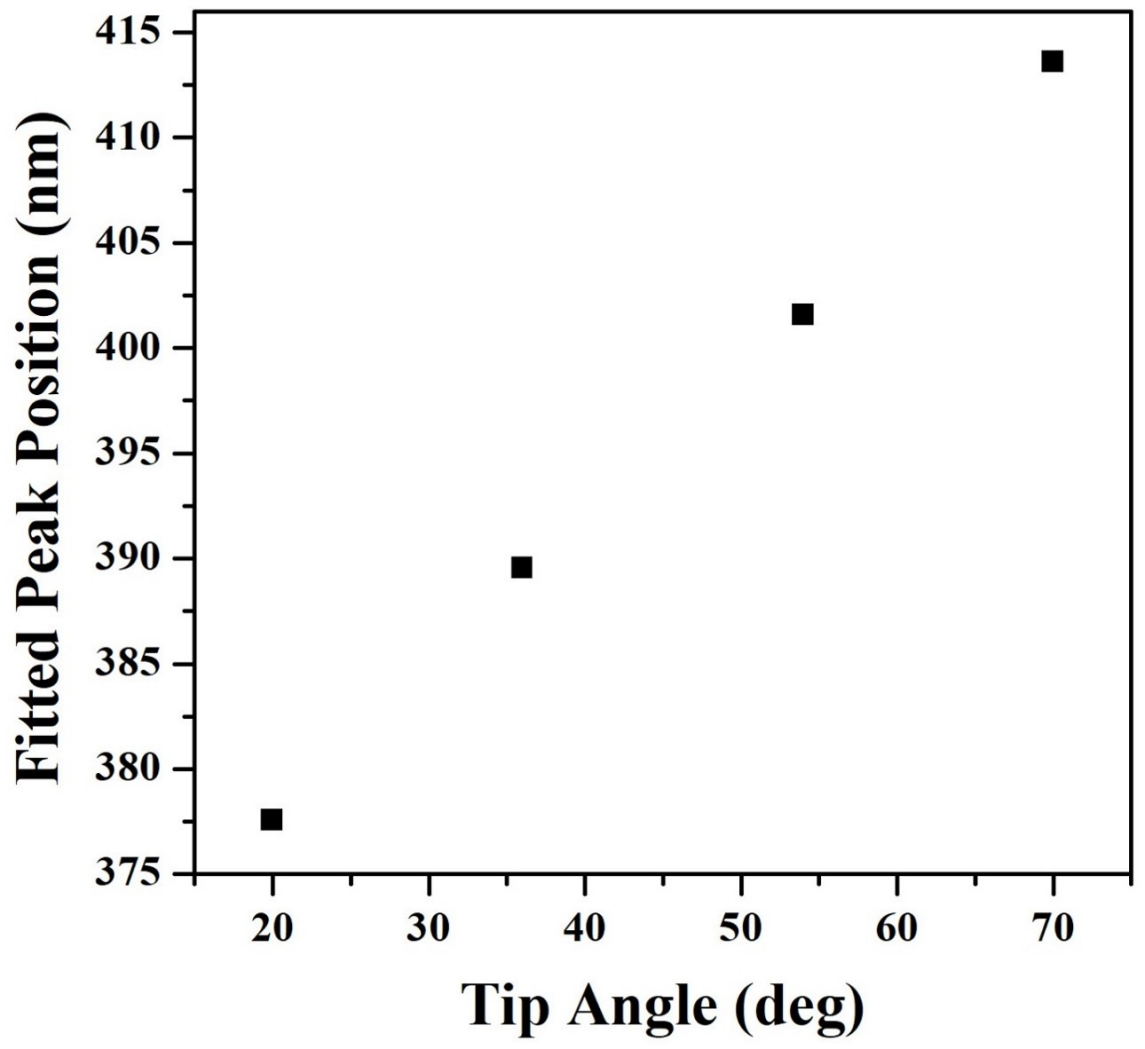
Fitted peak positions of tip plasmons from curves in Fig 5, respectively.

Fitted peak positions of tip plasmons are presented in Fig 6. The fitting results are presented in the supporting information (S3–S6 Figs). Tip plasmons exhibit a redshift when increasing the tip angle, which relates to the effective size of Bi_2_Te_3_ nanotips. This relationship agrees with the trend of other plasmonic nanostructures. As demonstrated in Ref. [37], the plasmonic emission wavelength (λ_sp_) of flat structures relates to d/n, where d and n are the effective size and the mode order. For our case, due to the existence of the metallic surface, the wavelength of tip plasmonic modes is in direct proportion to the confinement size at tip ends. As the decrease of tip angle, the structural size is smaller, and the corresponding wavelength of the standing tip plasmonic wave is shorter [38]. Meanwhile, when two edges are closer, the interaction between adjacent edges is getting stronger, so the resonant frequency of oscillating charges across boundaries is higher, leading to a shorter wavelength. Therefore, both methods demonstrates that the shift of plasmonic resonance is in direct proportion to the effective size (tip angle) of the structure.

**Fig 7:**
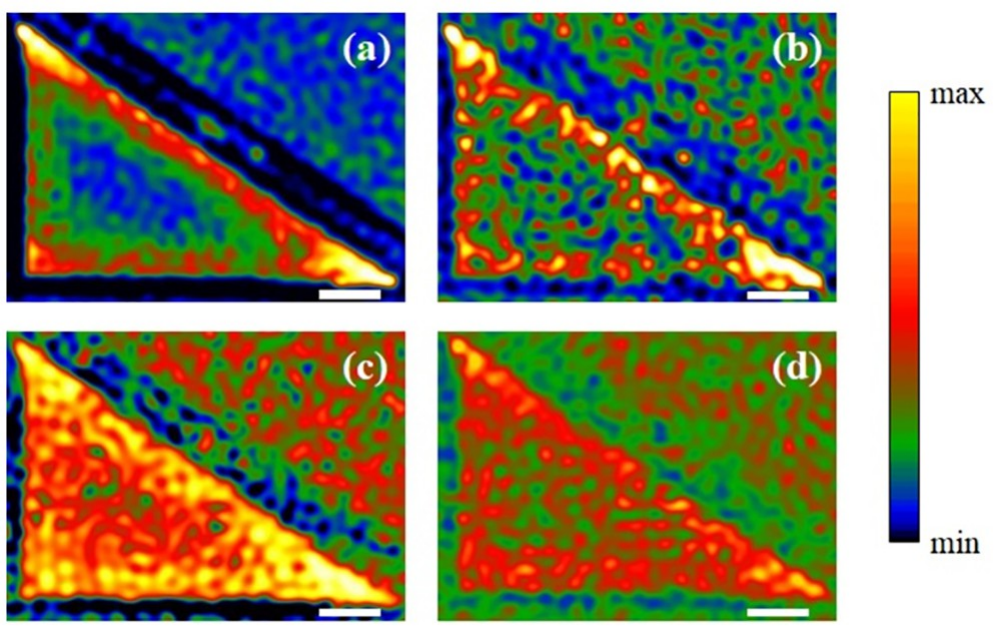
(a) Panchromatic CL image of the Bi_2_Te_3_ triangular nanoflake acquired by a PMT with a bandpass from 300 nm to 900 nm. (b-d) CL mappings of the same nanoflake acquired by a PMT with selective optical filters. Filters have an 80 nm bandpass, and a center wavelength of 400 nm, 500 nm, and 650 nm, respectively. All scale bars represent 1 μm.

CL panchromatic images provide an approach to directly visualize the integrated light emission when the beam is scanning across the entire sample surface. The CL panchromatic image of all emission from 300 nm to 900 nm can be determined in Fig 7(a). Three tips of the Bi_2_Te_3_ triangle are brighter than the center of the flake, and the two acute angles have prominent emission than any other area. Edges between any two tips obtain higher brightness than the center as well, corresponding to the edge plasmons caused by the resonance at boundaries [8]. Fig 7(b)–7(d) are the images acquired by PMT after selected by optical filters of 80 nm bandpass centered at 400 nm, 500 nm, and 650 nm, respectively. Although many possible SP modes could be included in the range of bandpass and overlap of detected wavelength may exist between images, different behavior of plasmonic emission have been observed in the wavelength selected CL maps. In Fig 7(b), the tip modes are dominant around 400 nm, generated by the enhanced plasmonic resonance with sharp angles. While in Fig 7(c), the photonic emission comes from all the surface, including the surface plasmon modes across the surface of the Bi_2_Te_3_ flake and the luminescence from the substrate. The photonic emission fades gradually at higher wavelength around 650 nm. The result of CL images is consistent with CL spectra, indicating that the plasmonic resonance of Bi_2_Te_3_ triangular antenna exists across the visible range. Sharp tips have a better ability to generate an enhanced plasmonic field.

## Conclusions

In summary, Bi_2_Te_3_ triangular antennas with distinct nano-tips have been designed for the investigation of plasmonic resonance. Then, Bi_2_Te_3_ nano-tips have been excited by localizing a high energy electron beam at the end of tips. The activated plasmonic emission can be collected and analyzed by a spectrally and spatially resolved CL system in the SEM work-station. An obvious comparison can be observed between the spectra from the tips and the background. Moreover, an enhanced tip-plasmon resonance is acquired from sharp tips. We demonstrate that the plasmonic property, especially the tip plasmon, of Bi_2_Te_3_ tips has a strong dependence on the size and excitation location on the tip. According to a better confinement for oscillating charges, sharp tips are able to be excited with a higher plasmonic energy. Our research provides a new route to fabricate and investigate plasmonic nanostructures of TIs in any size and geometry.

## Supporting information

S1 Fig Electron trajectories for the bulk sample from the cross-sectional view with a high magnification. Backscattered electrons and secondary electrons are shown in red and blue, separately.
(TIF)

S2 Fig Electron trajectories for the bulk sample from the cross-sectional view with a low magnification. Backscattered electrons and secondary electrons are shown in red and blue, separately.
(TIF)

S3 Fig Fitted curves of the spectrum from the 20° Bi_2_Te_3_ tip, with the fitted sub-peaks of tip plasmons (green), bulk/substrate emission (light blue), and edge plasmons (dark blue), respectively. The fitted peak position of tip plasmons is 377.56 nm.
(TIF)

S4 Fig Fitted curves of the spectrum from the 36° Bi_2_Te_3_ tip, with the fitted sub-peaks of tip plasmons (green), bulk/substrate emission (light blue), and edge plasmons (dark blue), respectively. The fitted peak position of tip plasmons is 389.57 nm.
(TIF)

S5 Fig Fitted curves of the spectrum from the 54° Bi_2_Te_3_ tip, with the fitted sub-peaks of tip plasmons (green), bulk/substrate emission (light blue), and edge plasmons (dark blue), respectively. The fitted peak position of tip plasmons is 401.58 nm.
(TIF)

S6 Fig Fitted curves of the spectrum from the 70° Bi_2_Te_3_ tip, with the fitted sub-peaks of tip plasmons (green), bulk/substrate emission (light blue), and edge plasmons (dark blue), respectively. The fitted peak position of tip plasmons is 413.61 nm.
(TIF)

S1 Data (ZIP)

S1 File (PDF)
