# Supplementary figures and images for: Cathodoluminescence and tip-plasmon resonance of Bi2Te3 triangular nanostructures
Source: PLoS One. 2024 Jan 19;19(1):e0291251. doi: 10.1371/journal.pone.0291251 (PMC10798455; doi:10.1371/journal.pone.0291251)

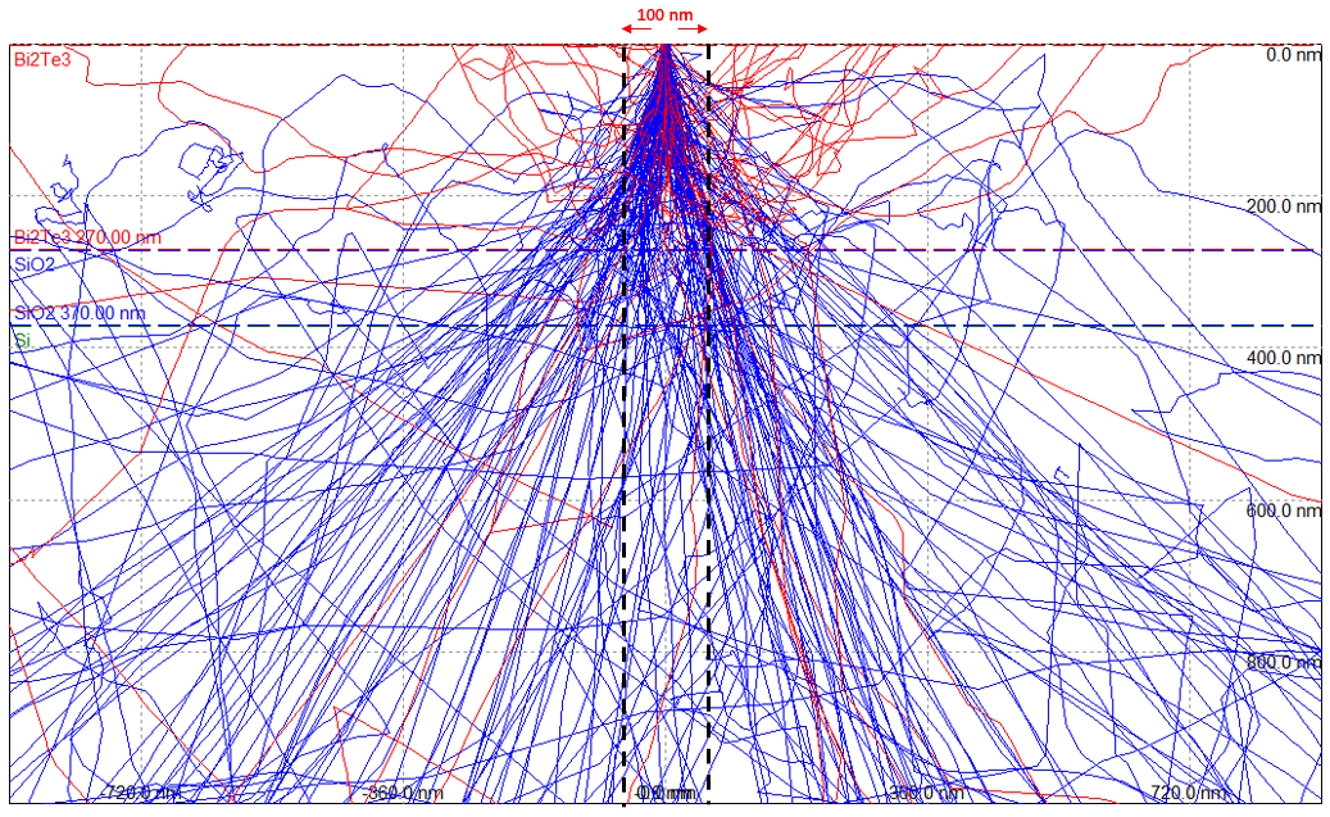

Supplement: S1 Fig — Backscattered electrons and secondary electrons are shown in red and blue, separately. (TIF) [file pone.0291251.s001.tif]

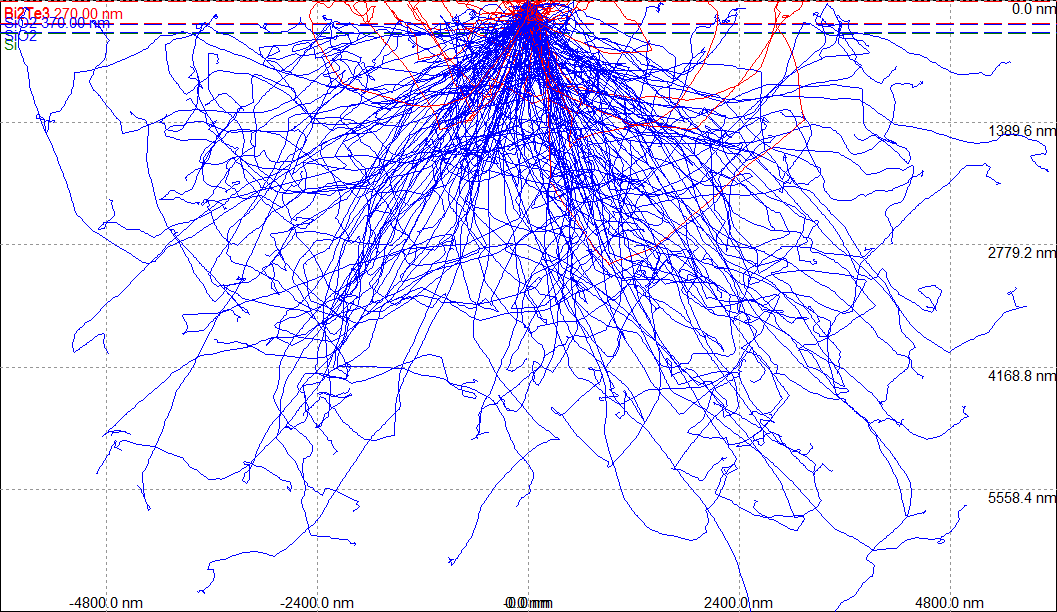

Supplement: S2 Fig — Backscattered electrons and secondary electrons are shown in red and blue, separately. (TIF) [file pone.0291251.s002.tif]

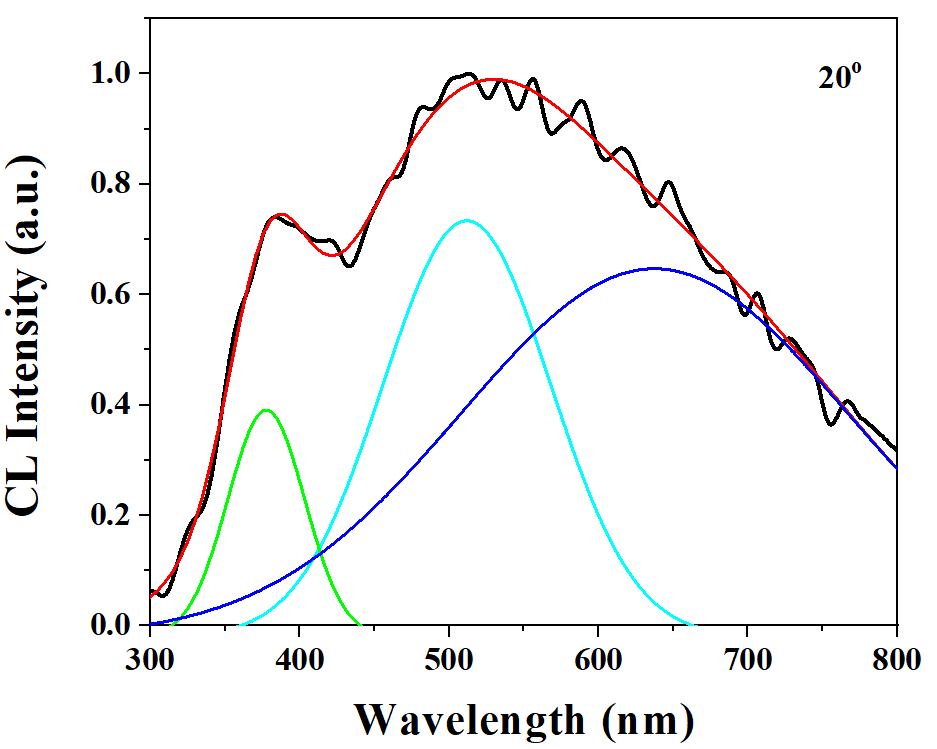

Supplement: S3 Fig — The fitted peak position of tip plasmons is 377.56 nm. (TIF) [file pone.0291251.s003.tif]

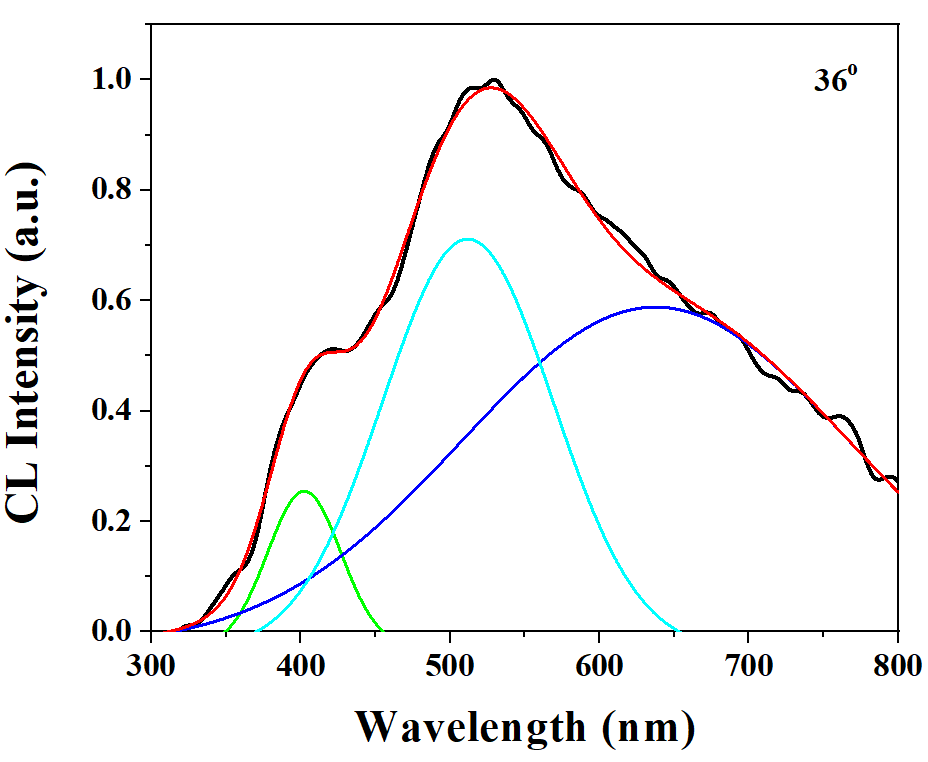

Supplement: S4 Fig — The fitted peak position of tip plasmons is 389.57 nm. (TIF) [file pone.0291251.s004.tif]

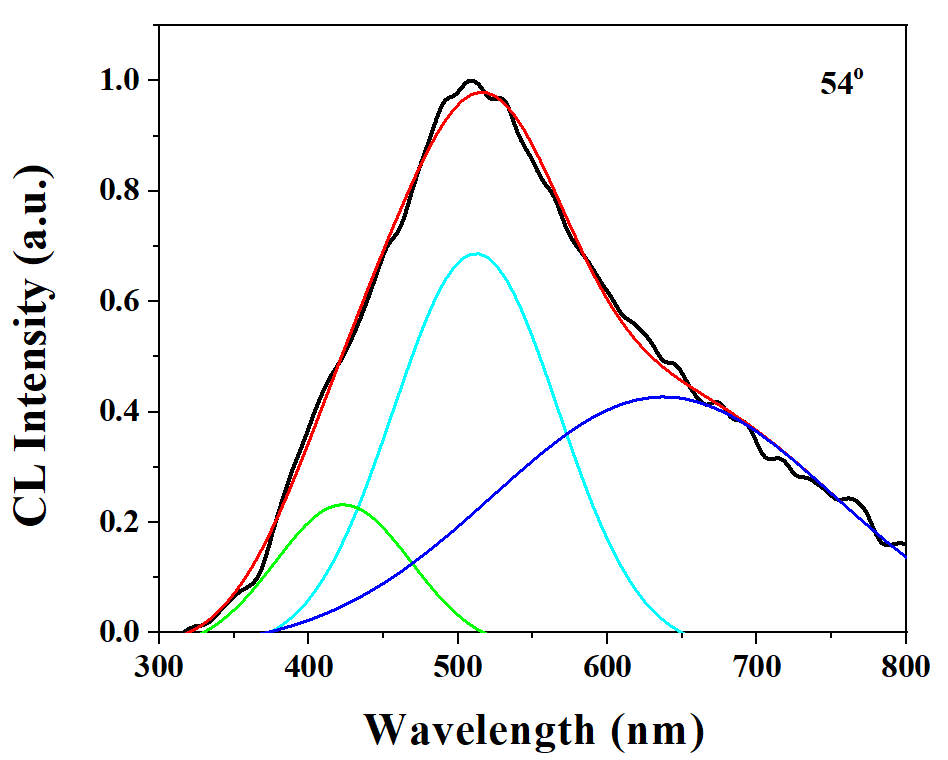

Supplement: S5 Fig — The fitted peak position of tip plasmons is 401.58 nm. (TIF) [file pone.0291251.s005.tif]

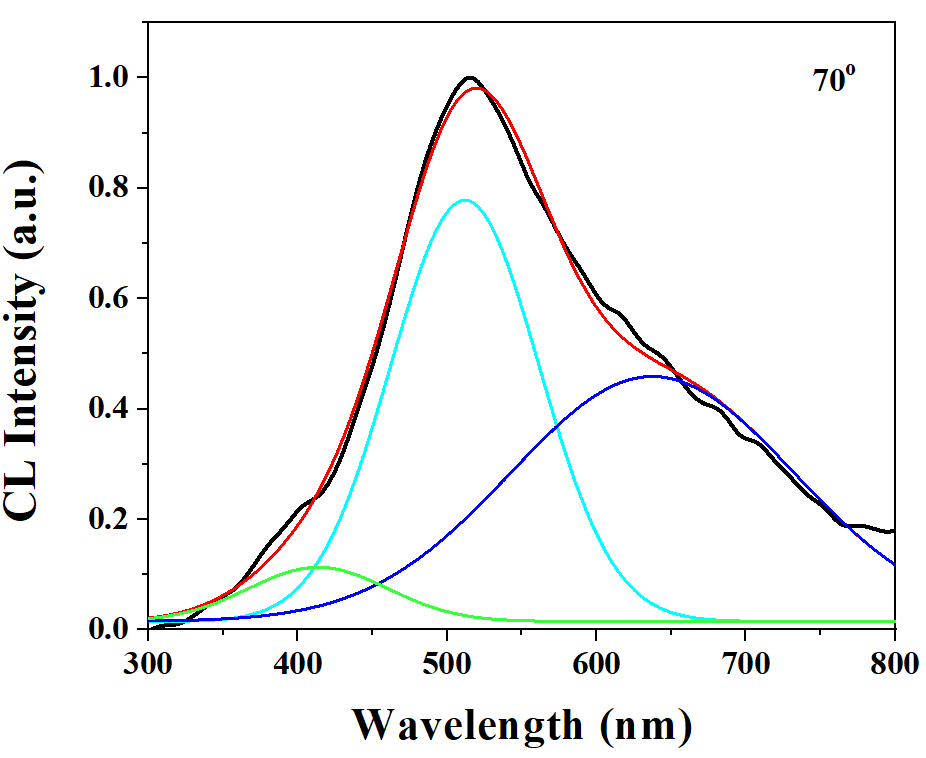

Supplement: S6 Fig — The fitted peak position of tip plasmons is 413.61 nm. (TIF) [file pone.0291251.s006.tif]
