# Supplementary material for: Cathodoluminescence and tip-plasmon resonance of Bi2Te3 triangular nanostructures
Source: PLoS One. 2024 Jan 19;19(1):e0291251. doi: 10.1371/journal.pone.0291251 (PMC10798455; doi:10.1371/journal.pone.0291251)
Supplement: S1 File — (PDF) [file pone.0291251.s008.pdf]

Supporting Information -- Cathodoluminescence and Tip-Plasmon Resonance of Bi_2Te_3 Triangular Nanostructures

Qigeng Yan [1,2,*], Siyuan Wang [3], Kuiwen Guan [2], Xiaojin Guan[2], Lei He[2]

1 Department of Physics, Baoding University, Baoding, Hebei, China

2 Department of Physics, University of Arkansas, Fayetteville, Arkansas, United States of America

3 Department of Science and Research, Baoding University, Baoding, Hebei, China

[*] Corresponding author

Email: yanqigeng@bdu.edu.cn

1. Monte Carlo Simulation of Electron Trajectories

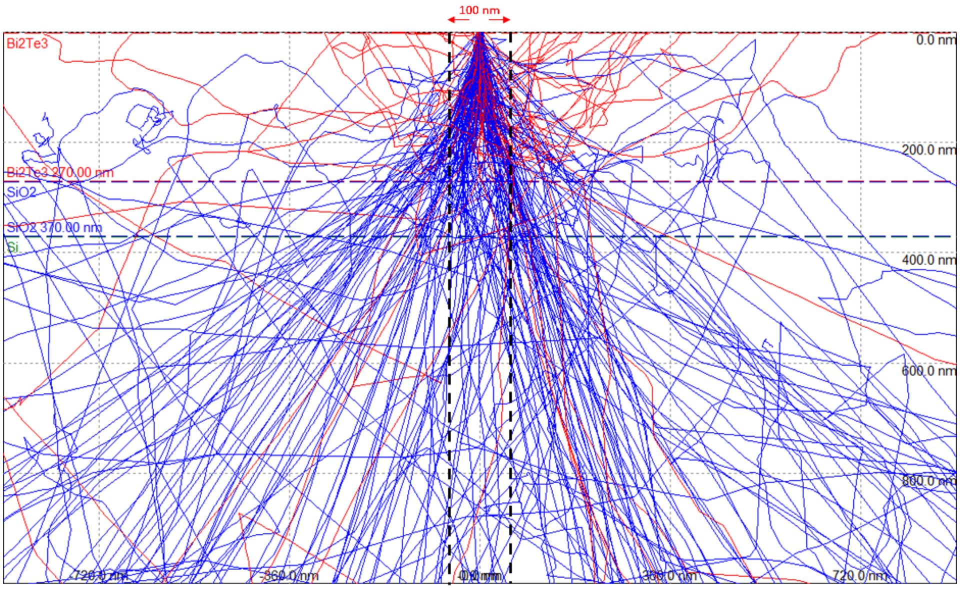

Figure S1. Electron trajectories for the bulk sample from the cross-sectional view with a high magnification. Backscattered electrons and secondary electrons are shown in red and blue, separately.

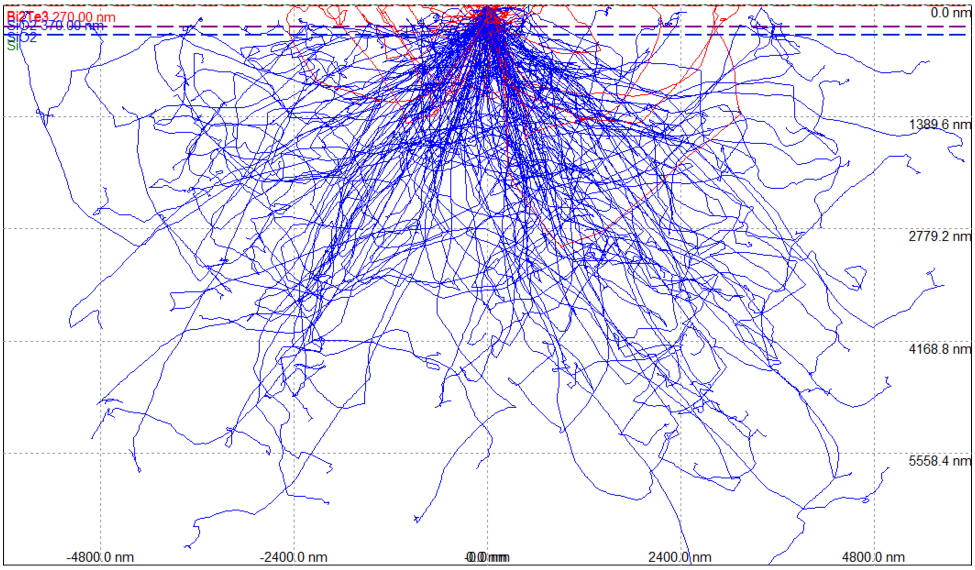

Figure S2. Electron trajectories for the bulk sample from the cross-sectional view with a low magnification. Backscattered electrons and secondary electrons are shown in red and blue, separately.

It is important to predict the interaction volume of electrons with samples before using cathodoluminescence (CL) for optical characterization. Therefore, the CASINO Monte Carlo simulation (ver 2.51, Dr. Drouin, University of Usherbrooke, Canada) for electrons is an effective tool to predict the trajectories of electrons before experiments, since the focused electron beam is applied for excitation in CL. Fig. S1 and Fig. S2 are showing cross-sectional simulations and the penetration area inside the sample. Blue lines represent the trajectories of secondary electrons, while red lines are the back-scattered electrons. Low energy secondary electrons at the shallow surface contribute to the excitation of surface plasmonic resonances. At the surface area, especially the first 100 nm depth, the broadening of e-beam is lower than 100 nm, which is smaller than most light sources. Some electrons may spread out and generate bulk plasmons or background emissions. Moreover, some electrons can penetrate direct into the Si substrate, indicating that CL spectra could include some signal from the deep area.

2. Line Fittings of Sharp Tips

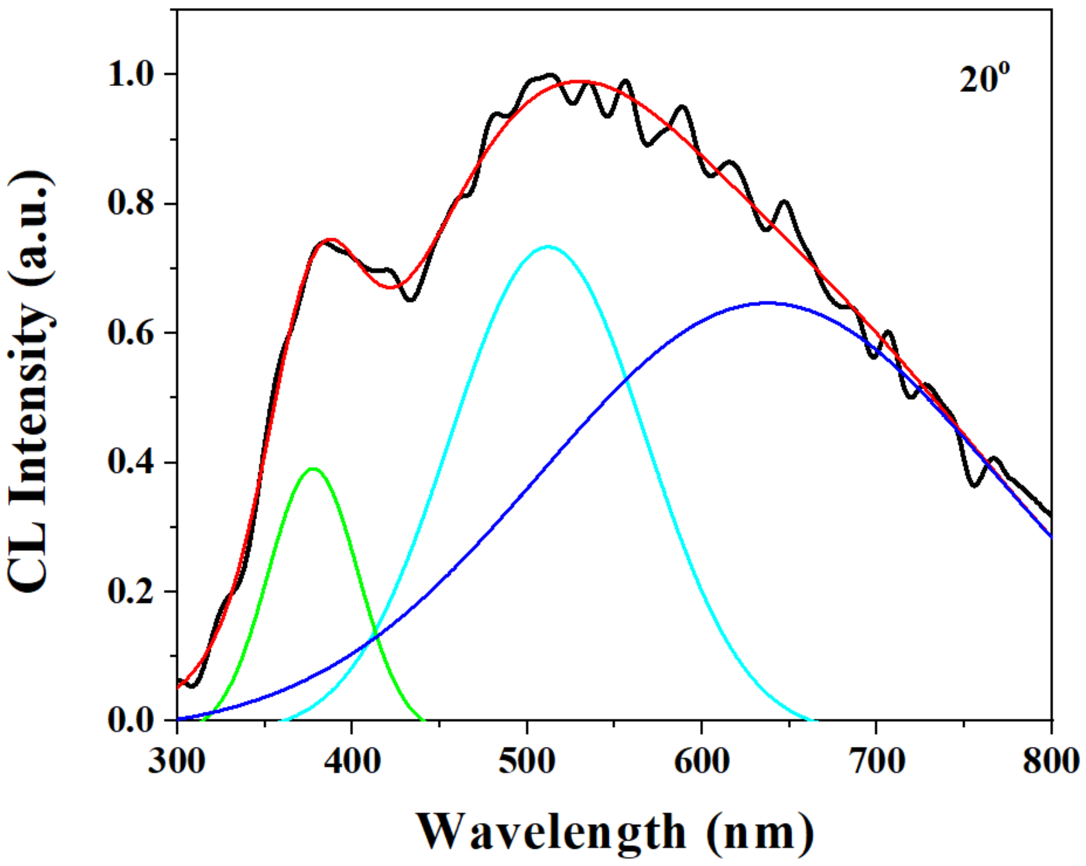

Figure S3. Fitted curves of the spectrum from the 20° Bi_2Te_3 tip, with the fitted sub-peaks of tip plasmons (green), bulk/substrate emission (light blue), and edge plasmons (dark blue), respectively. The fitted peak position of tip plasmons is 377.56 nm.

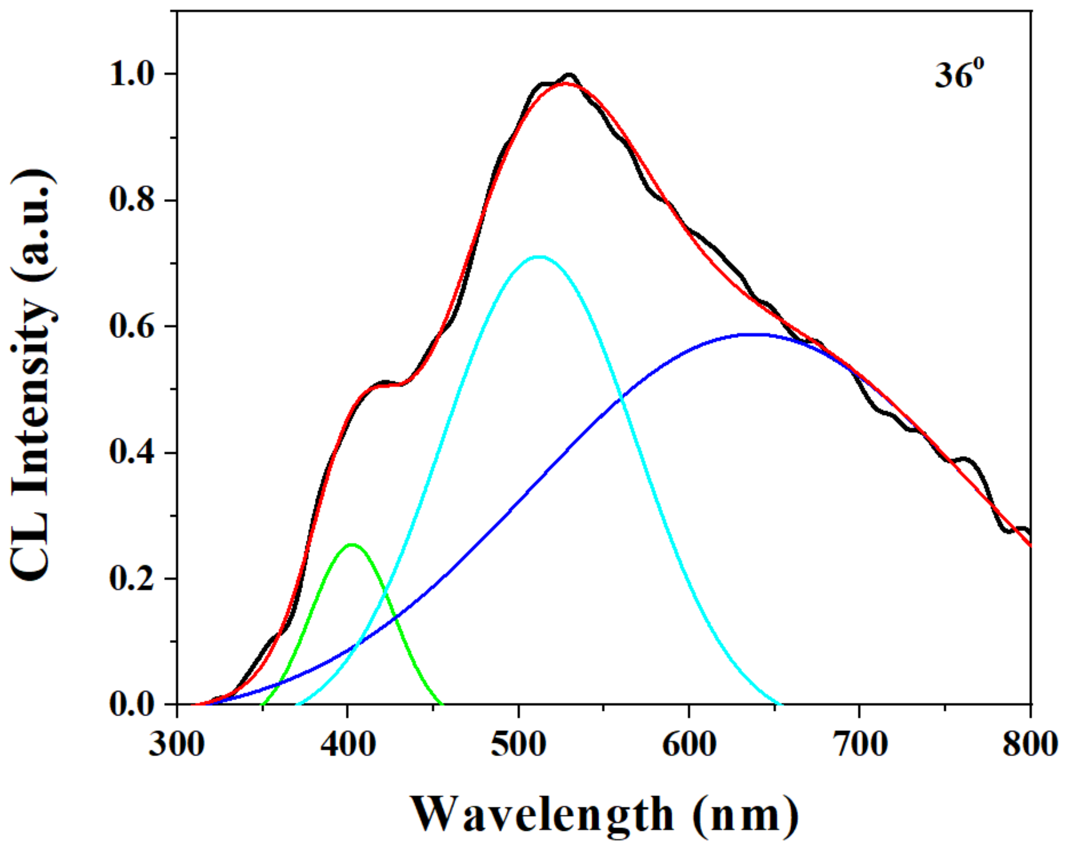

Figure S4. Fitted curves of the spectrum from the 36° Bi_2Te_3 tip, with the fitted sub-peaks of tip plasmons (green), bulk/substrate emission (light blue), and edge plasmons (dark blue), respectively. The fitted peak position of tip plasmons is 389.57 nm.

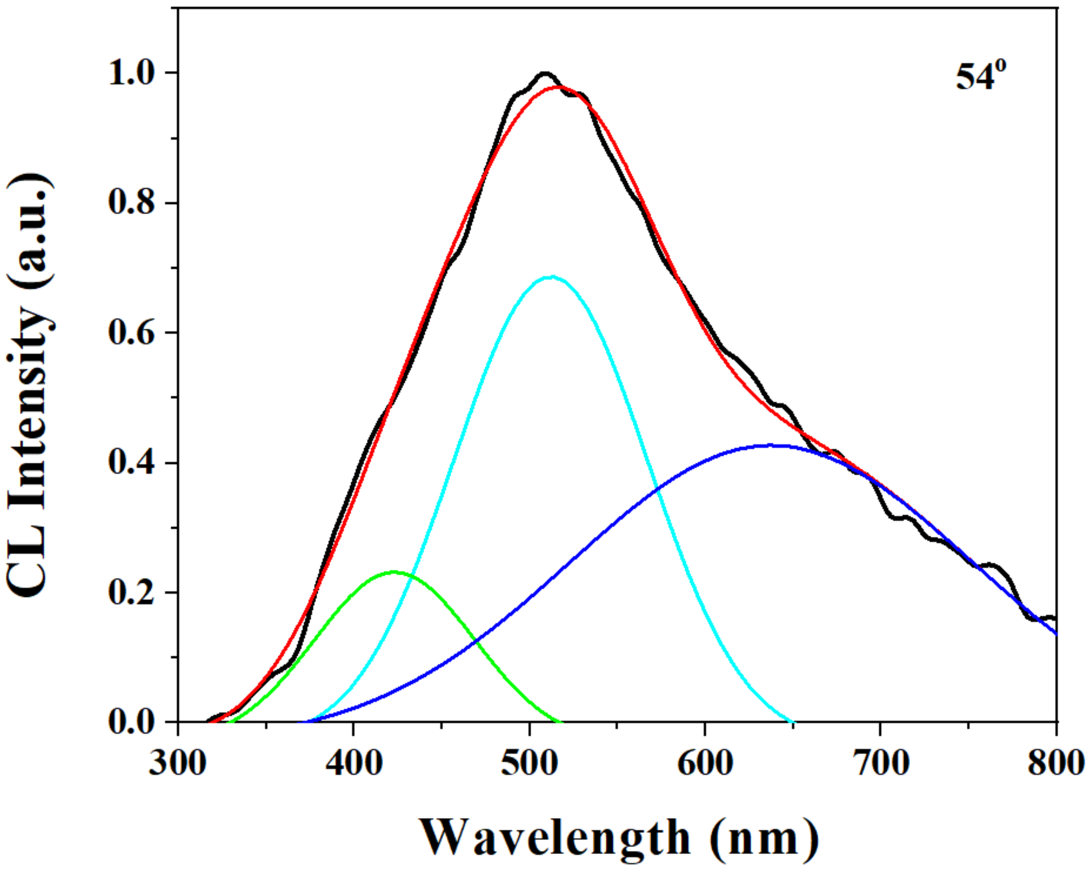

Figure S5. Fitted curves of the spectrum from the 54° Bi_2Te_3 tip, with the fitted sub-peaks of tip plasmons (green), bulk/substrate emission (light blue), and edge plasmons (dark blue), respectively. The fitted peak position of tip plasmons is 401.58 nm.

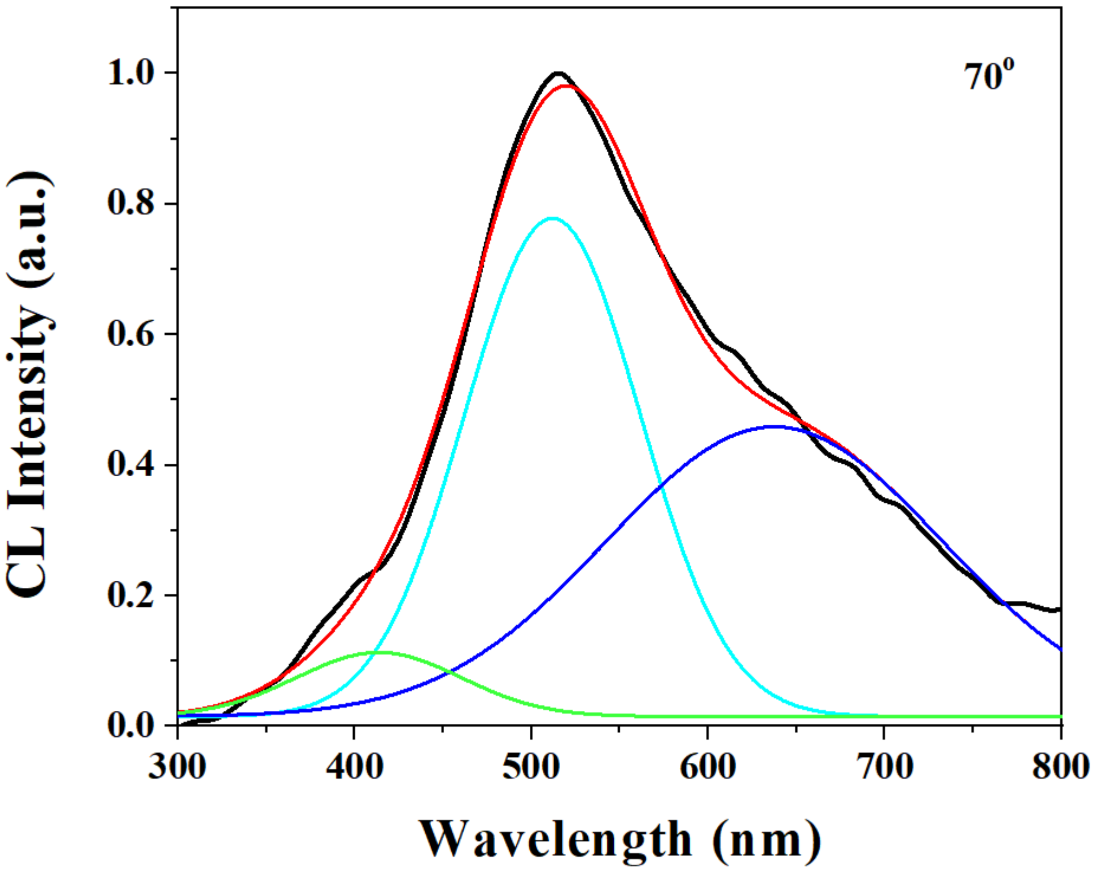

Figure S6. Fitted curves of the spectrum from the 70° Bi_2Te_3 tip, with the fitted sub-peaks of tip plasmons (green), bulk/substrate emission (light blue), and edge plasmons (dark blue), respectively. The fitted peak position of tip plasmons is 413.61 nm.

Fig. S3 to Fig. S6 are showing the fitted curves of CL spectra excited from sharp tips. The CL spectrum of the 90° tip has not been fitted, since it exhibits the similar line shape with the flake center and can be seen as the combination of the CL signal from the bulk and substrate. For fitted results shown above, the dark blue line stays in the long wavelength side, which is similar with the edge plasmon detected in Ref. 1. Therefore, the shoulder in the near infrared region relates to the collective plasmonic resonance at edges. The main peak, shown in light blue, relates to the background emission of the bulk material and substrate. This peak, around 510 nm, is dominant for all CL spectra, including all tips and the flake. When we confirm the origin of these two strong peaks, the last tip plasmon peak can be fitted, as shown in green. Meanwhile, the position of tip plasmonic peaks can be summarized, as demonstrated in Fig. 6 of the paper.

Reference:

[1] Zhao M., Bosman M., Mohammad D., Zeng M., Song P., Darma Y., et al. Visible Surface Plasmon Modes in Single Bi_2Te_3 Nanoplate. Nano Lett. 2015;15:8331-8335.
